# Identification of New Soluble Factors Correlated With the Development of Graft Failure After Haploidentical Hematopoietic Stem Cell Transplantation

**DOI:** 10.3389/fimmu.2020.613644

**Published:** 2021-01-29

**Authors:** Gerrit Weber, Luisa Strocchio, Francesca Del Bufalo, Mattia Algeri, Daria Pagliara, Claudia Manuela Arnone, Biagio De Angelis, Concetta Quintarelli, Franco Locatelli, Pietro Merli, Ignazio Caruana

**Affiliations:** ^1^Department of Pediatric Hematology/Oncology, Cell and Gene Therapy, Scientific Institute for Research and Healthcare (IRCCS), Bambino Gesù Childrens’ Hospital, Rome, Italy; ^2^Sapienza, University of Rome, Rome, Italy

**Keywords:** graft failure, cytokines, chemokines, inflammation, Th1 T cells, macrophage activation, hemophagocytic lymphohistiocytosis

## Abstract

Graft failure is a severe complication of allogeneic hematopoietic stem cell transplantation (HSCT). The mechanisms involved in this phenomenon are still not completely understood; data available suggest that recipient T lymphocytes surviving the conditioning regimen are the main mediators of immune-mediated graft failure. So far, no predictive marker or early detection method is available. In order to identify a non-invasive and efficient strategy to diagnose this complication, as well as to find possible targets to prevent/treat it, we performed a detailed analysis of serum of eight patients experiencing graft failure after T-cell depleted HLA-haploidentical HSCT. In this study, we confirm data describing graft failure to be a complex phenomenon involving different components of the immune system, mainly driven by the IFNγ pathway. We observed a significant modulation of IL7, IL8, IL18, IL27, CCL2, CCL5 (Rantes), CCL7, CCL20 (MIP3a), CCL24 (Eotaxin2), and CXCL11 in patients experiencing graft failure, as compared to matched patients not developing this complication. For some of these factors, the difference was already present at the time of infusion of the graft, thus allowing early risk stratification. Moreover, these cytokines/chemokines could represent possible targets, providing the rationale for exploring new therapeutic/preventive strategies.

## Introduction

One of the main complications occurring after allogeneic hematopoietic stem cell transplantation (HSCT) is represented by graft failure (GF). It is a complex and multifactorial syndrome characterized by hypocellular bone marrow (BM) associated with severe pancytopenia in peripheral blood (PB). GF can be defined based either on the pathophysiology mechanisms or on the timing of the event. Primary GF is characterized by lack of initial engraftment of donor cells, while secondary GF by the progressive loss of donor cells after initial engraftment. From a pathophysiological point of view, immune-mediated GF is caused by the attack of the donor cells by host immune cells, mainly T and Natural Killer (NK) cells surviving the conditioning regimen. Several factors have been reported to be associated with GF, including HLA disparity in the donor/recipient pair, presence of anti-HLA antibodies in the recipient, underlying disease, viral infections, type of conditioning regimen (particularly reduced-intensity conditioning and non-myeloablative conditioning), T-cell depletion of the graft (TCD) and stem cell source (1–4).

Our group has recently focused on a deep characterization of this phenomenon, analyzing a cytokine/chemokine asset in PB, (*i.e.*, IFNγ, sIL2Rα, CXCL9, CXCL10, TNFα, IL6, IL10, and sCD163), as well as the cellular features in BM biopsies of patients experiencing GF. From this analysis, we confirmed i) the *in vivo* role of the IFNγ-pathway in the development of immune-mediated GF; ii) that the sole inhibition of this pathway by an anti-IFNγ monoclonal antibody (mAb) was able to prevent GF. Finally, after observing a strong similarity between immune-mediated GF and hemophagocytic lymphohistiocytosis (HLH), we treated with Emapalumab, an anti-IFNγ mAb (5), on a compassionate use basis, three patients with primary HLH who, after having experienced GF, underwent a second successful HSCT.

In the present study, we tested other 44 cytokines/chemokines in the PB of the previously reported patients experiencing GF (5) with the aim of: i) further characterizing the GF signature; ii) identifying new possible targets to prevent/treat GF; iii) developing strategies capable to target a single pathway/molecule or a combination of them in order to prevent the occurrence of GF in patients at high-risk of developing this complication.

## Materials and Methods

### Patients and Controls

Children aged 0.3 to 21 years, given an allograft from any type of donor/stem cell source [including matched family donor (MFD), matched unrelated donor (MUD), unrelated cord blood unit (UCB), haploidentical family donor], between January 1, 2016, and August 31, 2017, at IRCCS Bambino Gesù Children’s Hospital in Rome, were considered eligible for the study. Patients or legal guardians provided written informed consent, and research was conducted under institutional review board approved protocols, in accordance with the Declaration of Helsinki. The Bambino Gesù Children’s Hospital Institutional Review Board approved the study.

After completing the main study (5), we performed further analyses on the remaining samples of 8 out of 15 patients experiencing GF after TCD haplo-HSCT and compared them with those of eight controls, matched for transplant characteristics, who had been transplanted reaching sustained donor engraftment during the same period.

### Cytokine Profile

Serum derived from patients experiencing GF and from a control group were analyzed by immunoassays incorporating magnetic microsphere technology (Merck, Darmstadt, Germany), according to the manufacturer’s instructions, as previously described (6). Plates were read on MAGPIX^®^ and analyzed using xPONENT^®^ software (Luminex, Austin, Texas, USA). The following cytokines and chemokines were analyzed: CCL1, CCL2, CCL3, CCL5 (Rantes), CCL7, CCL19, CCL20 (MIP3α), CCL24 (Eotaxin-2), CXCL11, CX3CL1, PDGFαα, CD40L, G-CSF, GM-CSF, FLT3-L, IL1α, IL1β, IL2, IL4, IL5, IL7, IL8 (CXCL8), IL9, IL11, IL12p40, IL12p70, IL13, IL15, IL17A, IL17E, IL17F, IL18, IL21, IL22, IL23, IL27, IL28A, IL31, IL33, SCF, and TNFβ.

### Statistical Analyses

Data are summarized as mean ± standard error of mean (SEM) and expressed as pg/ml. Student *t-*test (two-sided) was used to determine statistically significant differences between samples. When multiple comparison analyses were required, statistical significance was evaluated by a repeated measures ANOVA followed by a Log-rank (Mantel-Cox) test for multiple comparisons. P-values were reported in detail if statistically significant, i.e., <0.05 (*), <0.01 (**) and <0.001 (***). Graph generation and statistical analyses were performed using Prism version 7 software (GraphPad, La Jolla, CA). Interactome analysis on identified cytokines and chemokines modulated during GF was performed using STRING software (https://string-db.org) with a high interaction score (0.7).

## Results and Discussion

The samples of eight patients experiencing GF after receiving TCRαβ/CD19-depleted haploidentical HSCT (7) were compared to those of eight patients who did not develop this complication (during the study period we performed 115 haploidentical HSCT and 15 patients developed GF, the GF rate being 13%). Patient and control characteristics are detailed in **Table 1**. Main transplant characteristics were comparable between the two groups (except for a trend for a lower age in the GF group).

**Table 1:** Characteristics of patients and controls.

	**GF**	**CTRL**	**p**
**Total**	8	8	
**Gender**			0.99
Female	3	4	
Male	5	4	
**Age at transplant, years (median and range)**	2.4 (0.2–9.6)	7.0 (1.1–19.8)	0.08
**Disease**			0.37
PID	2§	1ç	
AL	1	4	
Hbpathies/IBMFS	2	2	
Others	3*	1#	
**Type of transplant**			0.2
T-cell depleted haploidentical	8	5	
MUD	0	3	
**Source of stem cells**			0.2
PBSC	8	5	
BM	0	3	
**Conditioning regimen**			0.43
TBI-based	0	1	
Busulfan-based	7	5	
Treosulfan-based	1	2	
**Sex mismatch**			0.99
Yes	2	3	
No	6	5	

^§^One case each of combined immunodeficiency and HLH.

^ç^One case of autosomal recessive hyper-IgE syndrome.

^*^One case each of metachromatic leukodystrophy, mucopolysaccharidosis type 1 and  osteopetrosis.

^#^One case of adrenoleukodystrophy.

PID, primary immunodeficiencies; AL, acute leukaemia; IBMFS, inherited bone marrow failure syndromes; MUD, matched unrelated donor: PBSC, peripheral blood stem cells; BM, bone marrow.

**Figure 1:**
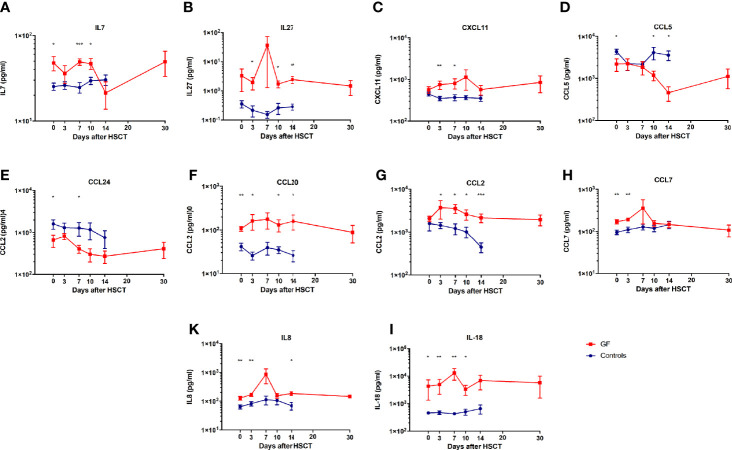
Cytokines and chemokines modulated during GF. Serum levels of IL7 **(A)**, IL27 **(B)**, CXCL11 **(C)**, CCL5 **(D)**, CCL24 **(E)**, CCL20 **(F)**, CCL2 **(G)**, CCL7 **(H)**, IL8 **(K)**, and IL18 **(I)** in patients who either did (red line) or did not (blue line) experience GF are shown. In all graphs mean and SEM for each variable are represented. * p<0.05, ** p<0.01, *** p<0.001.

We found a significant modulation of IL7, IL8, IL18, IL27, CCL2, CCL5 (Rantes), CCL7, CCL20 (MIP3a), CCL24 (Eotaxin2), and CXCL11 in patients experiencing GF (see **Figure 1**).

Interestingly, several of these molecules (IL7, IL8, IL18, CCL5, CCL7, CCL20, and CCL24) were significantly different from the control group already at the time of graft infusion (IL7: 47.8 ± 9.2 pg/ml vs. 24.2 ± 2.5; IL8: 127.5 ± 18.7 vs. 68.7 ± 10.3; IL18: 4334.6 ± 2993 vs. 468.8 ± 53.9; CCL5: 2188.3 ± 721.8 vs. 4148.8 ± 590.1; CCL7: 169.8 ± 19.2 vs 94.9 ± 11.3; CCL20: 108.1 ± 13.9 vs. 42.1 ± 8.2; and CCL24: 652.7 ± 217.8 vs 1426.5 ± 406.7). These findings suggest possible effects related to the conditioning regimen.

It is well known that the conditioning regimen can cause mild to severe tissue damage, which induces a production of several pro-inflammatory cytokines and chemokines from both hematopoietic cells, as well as by damaged endothelium and epithelia, increased expression of adhesion molecules, major histocompatibility complex antigens and costimulatory molecules on the host antigen presenting cells (APCs) (8). Host APCs, which survive the conditioning regimen, become activated and capable of processing antigens present in the transplanted cells. Activation of either recipient or donor T cells after interaction with host APCs leads to their proliferation, differentiation and migration.

The identified cytokines and chemokines underline the involvement of an inflamed microenvironment where T lymphocytes, NK cells, immature and mature APCs, among which monocytes and dendritic cells (DC), are recruited from the periphery to the BM (5). Several of these molecules are also able to sustain the inflammation and maintain activation of lymphocytes. In this context, our analysis reveals higher levels of IL7 (**Figure 1A**), which contributes to an inflamed BM microenvironment (9), sustains T-cell proliferation, differentiation and survival, in particular of the *naïve* and memory compartments (10), but also of mature differentiated T lymphocytes, through the Bcl2 pathway (11, 12). IL7 has also been reported to act as co-factor for T-cell activation by stimulating production of Th1 cytokines, including IFNγ, IL2, and TNFα (13). Moreover, in the allo-HSCT setting, high levels of these cytokines have been associated with graft-versus-host disease (GvHD) onset and its exacerbation, by either promoting proliferation and survival of allo-reactive donor mature T cells or by increasing their activation state (14). These data, associated with high levels of IL27, support the assumption of an activated environment, in particular in the BM niche (**Figure 1B**). This latter cytokine, indeed, is able to control both innate and adaptive immune responses by stimulating STAT3 (15, 16) and to block Th17 T-cell activity (17). Furthermore, it has been also associated with the development of GvHD, reducing the number of CD4^+^Tbet^+^ cells, increasing the number of CD8^+^Tc1^+^ cytotoxic T cells and inducing IFNγ response *in vivo* (18).

**Figure 2:**
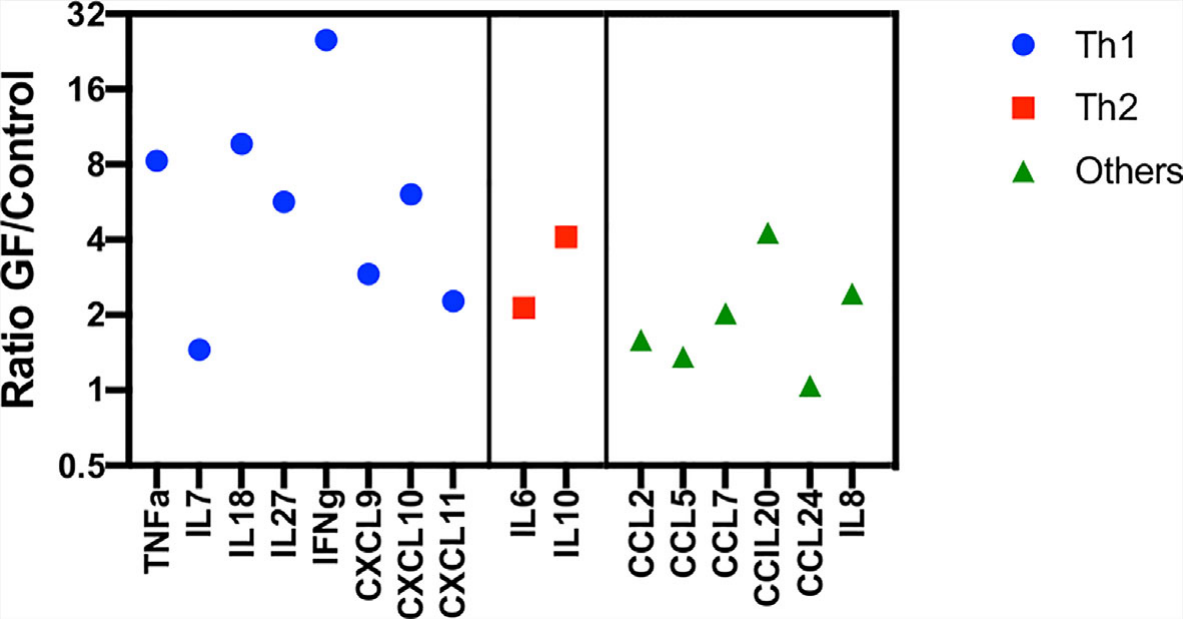
Cytokines/Chemokines found to be preferentially expressed in GF at day +3 after HSCT, grouped as “Th1,” Th2,” and “other.” Cytokine/chemokine levels are reported as ration between values measured in the GF and control group, respectively. This includes also cytokines/Chemokines previously reported in (5).

As reported in our previous study (5), this inflammatory state is mainly driven by IFNγ, which is able to activate macrophages and epithelia to produce CXCL9, CXCL10, but also CXCL11 (19) (**Figure 1C**). These chemokines are able to strongly recruit antigen-primed Th1 T cells directly to the inflamed tissue. Moreover, high levels of these cytokines have been associated with organ rejection in kidney, lung and heart transplantation (20–22). Furthermore, low levels of CCL5 and CCL24, like those found in present analysis, could, instead, be caused by a damage of endothelial and epithelial cells by activated and cytotoxic T lymphocytes, this translating into a further increase of the recruitment of Th1^+^ T cells expressing CXCR3 (**Figures 1D, E**). It is important to underline, however, that the ligands of CXCR3 (namely, CXCL9, CXCL10, and CXCL11) have been reported to be more potent than CCR5 ligands (i.e., CCL3, CCL4, and CCL5) and the frequency of CCR5^+^ T lymphocytes is significantly lower in PB circulating T cells (23, 24). The reduced levels of CCL5 can be also explained by the elevated conversion of monocytes into activated macrophages during this inflammation period (25, 26). As shown in **Figure 1F**, the macrophages present in the BM are able to produce high levels of CCL20 (MIP3α), which is actively involved in the recruitment of T lymphocytes and reported to be increased in renal graft rejection and, in general, during inflammation, causing a recruitment of mature DC (27–29). Our data emphasize the role of myeloid cells in boosting and maintaining inflammation: in fact, high levels of CCL2 and CCL7 underline the recruitment of monocytes, immature DCs, and macrophages together with effector T and NK lymphocytes (**Figures 1G, H**) (30–36). Furthermore, CCL2 has been also reported to play a crucial role in the M1 macrophage polarization during inflammation, in the recruitment of IFNγ^+^ γδ T cells and to regulate adhesion and chemotaxis through activation of β1 integrin and p38-MAPK (31, 37). In this altered microenvironment, we also detected high levels of IL8 and IL18 (**Figures 1K, I**). The first is physiologically produced by mononuclear cells and induces migration of lymphocytes to an injured site. High levels of this cytokine have been associated with GF, prolonged neutropenia and impaired differentiation of hematopoietic CD34^+^ cells (38). Its high expression has also been associated with increased levels of CCL2, CXCL9, CXCL10, and IL2Rα (39). Lastly, elevated levels of IL18 can be explained by an enriched IFNγ environment (40). The production of this cytokine, in fact, is mediated by the inflammasome and, in turn, it is responsible for sustaining IFNγ production in different lymphocyte subsets and is important for the differentiation of various T cell populations (40). Its accumulation has been associated with several immune-mediated diseases, including GvHD, and low overall survival of patients undergoing transplantations (41). IL18 is released by a damaged endothelium and is involved in macrophage activation, increasing expression of other pro-inflammatory cytokines (like CCL2) and in enhancing the activity of Th1 T and NK cells (42, 43). Its function is normally regulated by the presence of the high-affinity molecule IL18BP. For this reason, Liu et al. recently proposed to neutralize IL18 with IL18BP for the treatment of immune-mediated conditions, in which injury-associated cytokines are produced, including IFNγ and CXCL10 (44). In support to the probable role of macrophages and endothelial damage in the development of GF, recently, IL18 has been also described as potential biomarker and therapeutic target of macrophage activation syndrome/HLH, which shares, as mentioned before, several important features with GF (45). Notably, after grouping cytokines analyzed in this and in our previous study (5) as Th1, Th2, or “others,” the Th1 profile seems to be predominant (**Figure 2**), although contra-regulatory Th2 cytokines (in particular IL10) are increased (as already reported other hyper-inflammatory conditions, such as in primary HLH (46)).

We acknowledge that, beside the limited sample size, the lack of samples collected before the conditioning regimen represents a limitation of the study, preventing the evaluation of its influence on the cytokine “signature” at time of transplant. Moreover, although not statistically significant, some differences in the conditioning regimens used may have influenced the cytokine profile. Additionally, since one patient in the GF group was affected by HLH, this could impact the cytokine profile of this individual subject (more in general, patients with primary immunodeficiencies may have altered cytokine production). Finally, we acknowledge that infections may influence the pattern of cytokine production. In this regard, the cumulative incidence of bacterial, viral and fungal infection was similar in the two groups investigated (see **Table 2** and **Supplementary Figure 1** for details). For this reason and given that the differences in cytokine levels were already present at very-early time-points, it is unlikely that this factor has influenced the cytokine profile of GF patients and controls.

**Table 2:** Details on infections recorded in the GF and control cohorts during the study period.

	GF	CTRL
**Total**	4	3
**Viral**	4*	**1**
CMV	3*	1
Adv	1*	
HHV6	1	
**Bacterial**	**0**	**2**
*E. faecium*		1
*S. capitis*		1
**Fungal**	0	0

*One patient developed a coinfection with CMV and Adv.

**Figure 3:**
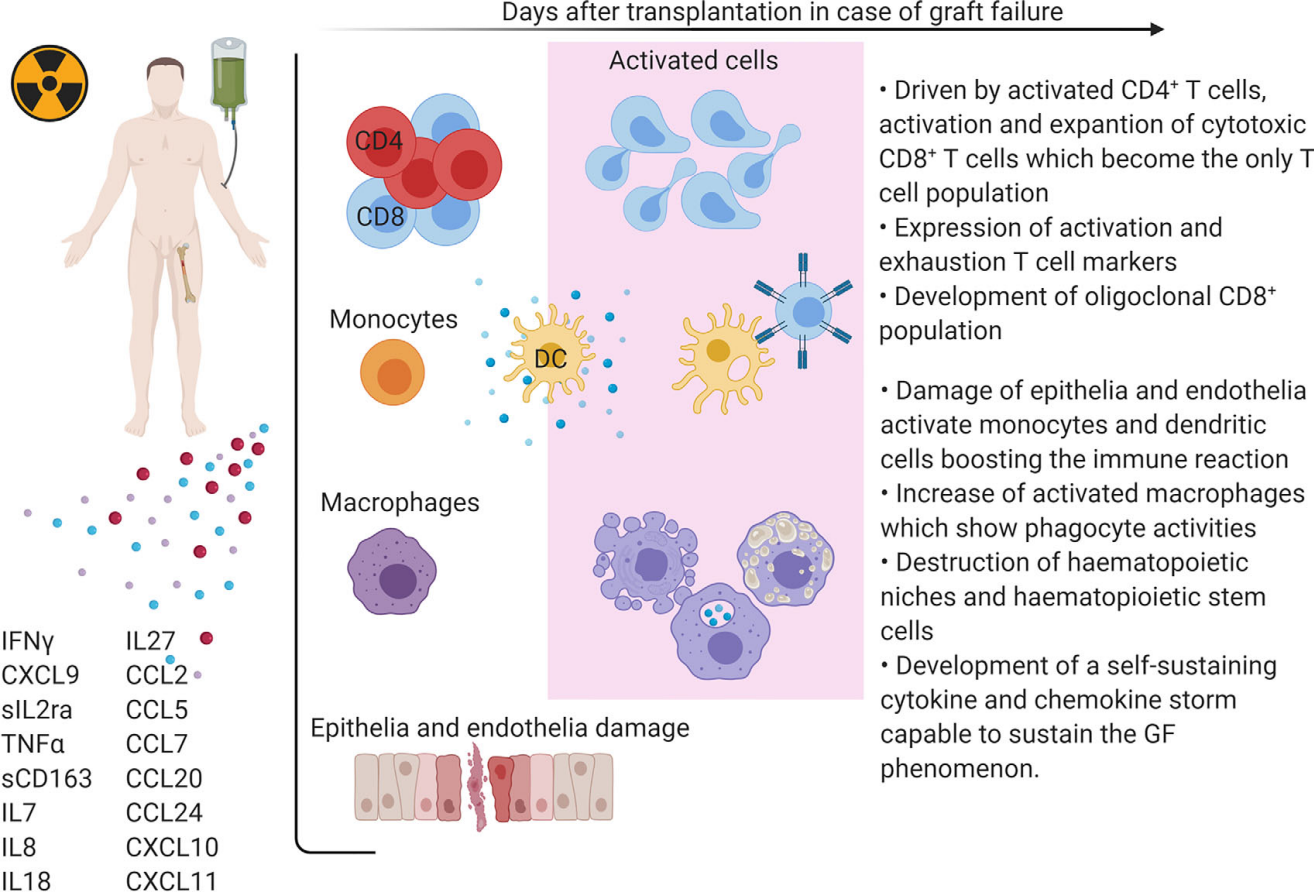
Schematic representation of GF pathophysiology after HSCT.

**Figure 4:**
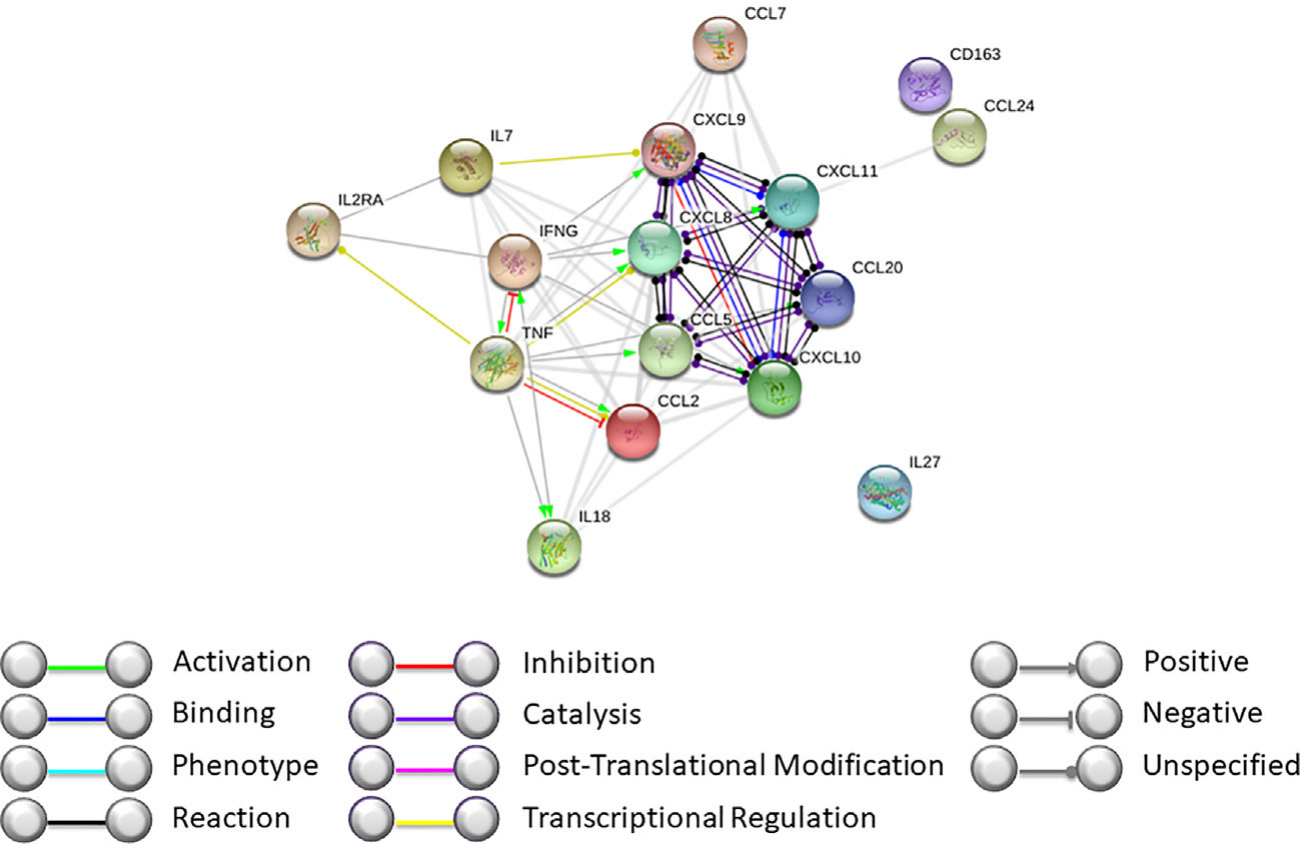
Interactome analysis on identified cytokines and chemokines modulated during GF was performed using STRING software (https://string-db.org). Interactome of cytokines and chemokines modulated during GF after HSCT with high confidence score.

Our data, together with those previously published by our group, support the hypothesis that during GF, complex mechanisms are activated and involve both soluble molecules and cellular components (**Figures 3** and **4**). By interactome analysis performed using STRING algorithm, several of these molecules were shown to be critical for the triggering and sustaining the pathophysiology of GF (**Figure 3**). Based on these data, strategies to prevent and treat this life-threatening complication can be considered. Notably, the use of emapalumab, a humanized mAb that binds and neutralizes IFNγ, currently approved for the treatment of adult and pediatric patients with primary HLH with refractory, recurrent or progressive disease or intolerance with conventional HLH therapy (47), has been explored as compassionate use (5, 48). Moreover, inhibition of cytokines like IL18 or IL27, as well as strategies aimed at compensation of the microenvironment increasing Th2 cytokines and chemokines (IL1β and CCL24), can be hypothesized.

## Data Availability Statement

The raw data supporting the conclusions of this article will be made available by the authors, without undue reservation.

## Ethics Statement

The studies involving human participants were reviewed and approved by Ethics Committee of Bambino Gesù Childrens’ Hospital. Written informed consent to participate in this study was provided by the participants’ legal guardian/next of kin.

## Author Contributions

IC, PM, and FL designed the study and analyzed the data. LS, FD, MA, DP, BD, CQ, and PM treated patients, collected samples and data, analyzed data, and edited the paper. GW, CMA, and IC performed immunomagnetic assays, analyzed data, and wrote the paper. All authors contributed to the article and approved the submitted version.

## Funding

This work was partly supported by grants from: Ministero della Salute (“Ricerca Corrente” to IC and PM) and AIRC (Associazione Italiana Ricerca sul Cancro, Investigator Grant—ID 21724—and Special Program Metastatic disease: the key unmet need in oncology 5 per mille 2018—ID 21147—to FL).

## Conflict of Interest

PM and FL have received honoraria from SOBI.

The remaining authors declare that the research was conducted in the absence of any commercial or financial relationships that could be construed as a potential conflict of interest.

## Supplementary Material

Supplementary Figure 1 Cumulative incidence of infections (bacterial, viral and fungal) in GF patients and controls.
