# Supplementary figures and images for: Identification of New Soluble Factors Correlated With the Development of Graft Failure After Haploidentical Hematopoietic Stem Cell Transplantation
Source: Front Immunol. 2021 Jan 29;11:613644. doi: 10.3389/fimmu.2020.613644 (PMC7878541; doi:10.3389/fimmu.2020.613644)

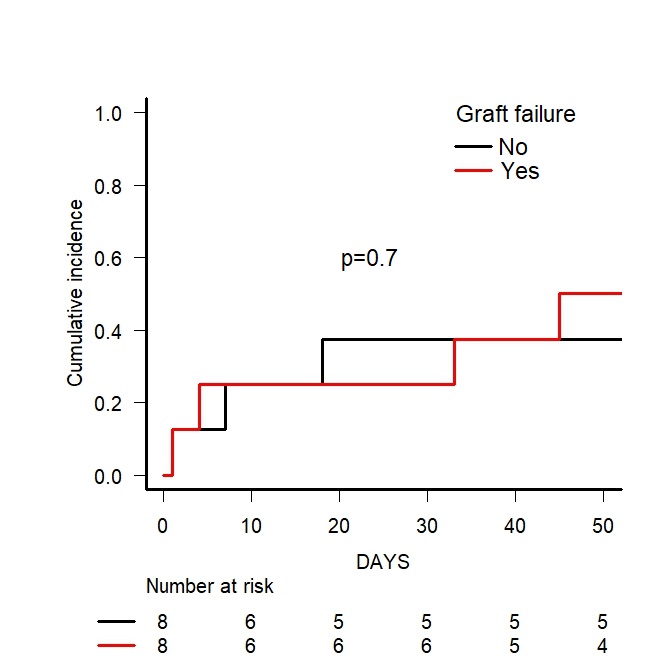

Supplement: Supplementary Figure 1 — Cumulative incidence of infections (bacterial, viral and fungal) in GF patients and controls. [file Image_1.jpeg]
